# A Research-Informed Approach to Providing Behavioral Healthcare to Women with Extensive Trauma Histories

**DOI:** 10.1007/s10597-023-01159-1

**Published:** 2023-07-13

**Authors:** Alison Greene, Josephine D. Korchmaros

**Affiliations:** 1 University of Arizona, Southwest Institute for Research on Women, 925 N Tyndall Avenue, P.O. Box 210438, Tucson, AZ 85721-0438 USA; 2 School of Public Health-Bloomington, Indiana University, 1025 East 7th Street, Bloomington, IN 47405-7109 USA

**Keywords:** Trauma, Substance use, Homelessness, Mental health, Treatment implications, Research to practice

## Abstract

Translating research to behavioral healthcare practice is vital for improving treatment impact but can be challenging. Current and lifetime histories of trauma need to be considered in behavioral healthcare provision as they can significantly affect an individual’s treatment experience. This article provides guidance on how to utilize research findings regarding trauma prevalence and experiences of women who have substance use disorder and who are homeless or near homeless to help guide responsive healthcare and treatment in practice.

Translating research to inform and strengthen behavioral healthcare, including mental healthcare and substance use disorder (SUD) treatment, is critical to improving its positive impact. Yet, it is often challenging to operationalize or utilize research findings in practice (Higa & Chorpita, 2008; Hutchinson & Johnston, 2006). High rates of trauma experience and history among different populations underscore the necessity of implementing a trauma-informed approach to treatment (Substance Abuse and Mental Health Services Administration [SAMHSA], 2014). Responding to lived experiences and population needs may increase sustained engagement in affirming behavioral healthcare treatment and services and, as a result, may increase treatment effectiveness (Hales et al., 2019; Palmieri & Valentine, 2021). One specific population with high rates of mental health, behavioral health, and substance use disorder (SUD) treatment needs is women who have SUD and who are homeless or near homeless (Polcin, 2016; Upshur et al., 2017). When providing behavioral healthcare to this population, it is critical to consider their trauma history as it can significantly impact their treatment experiences (SAMHSA, 2014). This article provides guidance on how to utilize information in the literature regarding trauma prevalence and experiences of women who have SUD who are homeless or near homeless to help guide responsive healthcare and treatment in practice.

## Research-Informed Strategies for Providing Trauma-Informed Care to Women Who have SUD and Any History of Homelessness

Greene and colleagues (2023) present a trauma experience profile of women who have SUD who are homeless or near homeless. They assessed seven types of trauma experience, including sexual abuse and assault; physical abuse and assault; non-interpersonal threat to physical health; forced displacement; traumatic grief and separation from children; drug partners and family; exposure to community violence; and justice involvement. They found that all of the women in their study had experienced at least one of the seven types of trauma experiences in their lifetime. Over 75% of the participants experienced five to seven of the types of trauma experiences assessed, and 20% had experienced all seven types of trauma during their lifetime. The participants reported high levels of emotional severity related to the majority of traumatic events experienced.

This profile provided by Greene and colleagues (2023) can be useful to informing the approach to providing behavioral healthcare treatment and services, and considerations for the clinical experience. Implementing a research-informed approach to direct trauma-informed care in mental health, SUD, and broader behavioral health treatment settings can improve both the experience and outcomes for those seeking clinical care. Treatment organizations can consider implementing strategies at both the organizational- and the clinical-level to increase trauma-informed care that is responsive to the specific trauma histories of women who have SUDs and are homeless or near homeless.

Ideally, this would start with an organizational commitment such that the clinical practices of providing trauma-informed care are supported within the broader culture of a trauma-informed health care setting (Menschner & Maul, 2016). At the larger organization-level this would include recognizing the impact of trauma and prioritizing patient empowerment in organization approach and decision-making by hiring a trauma-informed workforce; training non-clinical as well as clinical staff in trauma-informed practices; creating a safe organizational environment; and preventing secondary traumatic stress in staff. At the clinical level, there are meaningful trauma-informed care strategies, which include screening for trauma and tailoring services to meet individual needs; involving patients in the treatment process; training staff in trauma-specific treatments; and engaging referral sources and partnering organizations to meet the individual needs of patients.

### Organizational-Level Strategies

#### Having a Trauma-Informed Workforce

Based on the high prevalence and extensiveness of the trauma histories of women with SUDs who are homeless or near homeless (Greene et al., 2023), it is critical for organizations serving this population of women to employ staff that have training in trauma-informed care and experience with high-trauma history populations. If there are limitations in hiring candidates with this experience and education, assessing candidates for characteristics such as empathy, acceptance, and non-judgmentalism can be a meaningful strategy for building an organizational culture of trauma-informed care (Menschner & Maul, 2016).

Multiple types of employees comprise mental health, behavioral health, and SUD treatment organizations. Many of these employees are non-clinical staff such as schedulers, front desk workers, administrators, security guards, and care coordinators. Women with SUDs who are homeless or near homeless and accessing treatment in various settings will often interact with these non-clinical staff members before and more frequently than they do with clinical staff. These non-clinical staff also greatly contribute to setting the tone of the organization’s environment as they are often positioned in and near the site’s entrance and waiting area. Yet, the impact these staff have on engaging and retaining patients is often overlooked (Menschner & Maul, 2016). There is great opportunity to include non-clinical staff in trauma-informed care trainings to reinforce that all staff contribute to creating a trauma-informed organizational culture.

#### Creating a Safe Organizational Environment

A safe physical organizational environment is critical for engaging and retaining women who have a history of trauma, such as those who have SUD and are homeless or near homeless. Considerations such as well-lit parking areas and entrances, welcoming signage, and clear access to exits can create feelings of safety in the physical environment of the organization. Feeling unsafe in a physical environment may trigger patients with histories of trauma and may potentially be retraumatizing. It is particularly important to be thoughtful about all aspects of the treatment experience for women who are homeless/near homeless and have SUD. Women with histories of trauma also need to feel socially and emotionally safe in the organizational setting. Strategies that can be utilized include welcoming patients; ensuring all staff maintain healthy boundaries; treating each patient with respect, compassion, and support; and creating an environment that is responsive to the diversity of the community in which the organization is housed (Menschner & Maul, 2016). Prioritizing physical and emotional safety within mental health, behavioral health, and SUD treatment organizations will decrease barriers for women with SUDs who are homeless or near homeless to access needed treatment.

#### Preventing Secondary Traumatic Stress in Staff

Many women in the study by Greene and colleagues (2023) reported traumatic stress and related experiences, such as sexual assault, physical assault, and suicidal thoughts, that may be distressing for staff who work with this population. Secondary traumatic stress, which is the emotional strain caused by listening to an individual’s trauma experience, is a natural but disruptive consequence of working with and helping highly traumatized populations (Figley, 1995; Osofsky et al., 2008). Clinical and non-clinical staff experiencing secondary traumatic stress may face difficulties in providing effective patient care. In addition, over time, the impact of the secondary traumatic stress may lead to burnout. Leadership and administrators of mental health, behavioral health, and SUD treatment organizations should consider strategies to prevent secondary traumatic stress among staff. These strategies can include, for example, encouraging and incentivizing self-care activities, allowing staff to take time off for mental health, providing trainings to raise awareness of secondary traumatic stress, and providing support through regular supervision to process feelings that arise in response to patient care. Caring for the organizational staff will reduce the burden of secondary traumatic stress, which, in turn, may reduce staff burnout and potentially staff turnover (Menschner & Maul, 2016).

### Clinical-Level Strategies

#### Screening for Trauma and Tailoring Services

Descriptions of trauma profiles of different population provided in the literature, such as provided by Greene and colleagues (2023), contribute to clinicians’ general understanding of the populations and inform the tailoring of services to ensure that services and the provision of them are trauma-informed. However, an evidence-based screening tool can provide specific information to further tailor services to meet the needs of the individual a clinician serves. However, the process of screening comes with risk. For example, women with extensive trauma histories may experience the screening as retraumatizing, which would be directly contrary to providing trauma-informed care. Thus, the benefit of and approach to screening should be carefully considered and balanced against the possible harm of the screening procedure to the patient and their experience. Clinicians administering the screening tool should be proficient in how to conduct a trauma-informed screening and related interactions should be culturally appropriate and supportive. The screening should benefit both the woman being served as well as the clinician in order to provide high-quality coping strategies and treatment that are responsive to the woman’s specific needs. In sum, screening for specific trauma can be a proactive approach to effectively tailoring services and providing care in mental health, behavioral health, and SUD treatment settings, however potential risk related to re-traumatization should be considered.

#### Involving Patients in the Treatment Process

A trauma-informed approach recognizes the need for patients to be actively engaged in their treatment and values patient input to help direct the treatment plan. During experiences of trauma, women often have their choice taken away and their voice silenced (Wilson et al., 2015). Involving patients in their own treatment process is a deliberate way to empower them to have control over decisions and raise their voice. Some strategies include presenting patients with different treatment options and letting them dictate the degree to which there is collaboration among treatment providers, family members, and themselves. Another strategy with potential for positive impact is the use of peer coaches because interactions with a trained individual with lived trauma experiences may lead to greater trust and improved care. Utilizing a peer coach or peer support model can also encourage and build skills such as leadership, positive social interaction, and positive coping.

#### Training Staff in Trauma-Specific Treatments

As the field of trauma-informed care has grown, clinicians and patients have benefitted from the emergence of evidence-based trauma-specific treatment interventions. For example, Seeking Safety (Najavits, 1999, 2007) is a trauma-informed evidence-based treatment to address co-occurring SUD and traumatic stress-related needs. Studies have evidenced positive outcomes of Seeking Safety on SUD and post-traumatic stress disorder (PTSD) for women in a variety of treatment settings (Najavits & Hien, 2013; Zlotnick et al., 2003). Another example is Helping Women Recover, which is an evidence-based program for treating addiction that integrates theories of women’s psychological development, trauma, and addiction to specifically meet the needs of women (Covington, 2000). It has been shown to result in reductions in substance misuse and arrest, and better in-treatment experiences with more positive perceptions of the treatment experience in women compared to women who received mixed-gender treatment (Messina et al., 2012). Regardless of which evidence-based trauma-specific treatment intervention an organization selects, training staff in these specific models and approaches is necessary for serving women who have SUDs and are homeless or near homeless as their trauma experience is extensive (Greene et al., 2023).

Among the evidence-based trauma-specific interventions available, some are designed for individual or one-on-one treatment delivery and others for delivery in a group format. When providing treatment in a group format, it is important to avoid creating opportunities for women who have a trauma history to be retraumatized. This can occur by listening to a peer in the group talk about her trauma experiences or if a participant shares maladaptive coping strategies that could trigger or have a negative impact on another group member. In consideration, treatment delivery mode as well as training staff to deliver trauma-informed interventions with fidelity are critical components of trauma-informed service provision.

#### Engaging Referral Sources

Women with SUD who are homeless or near homeless will present with multiple needs. Often these needs are best met through a coordinated effort involving multiple partnering organizations. Thus, it is imperative to foster relationships with referral sources and other organizations that value and respect the degree to which trauma impacts the lives of those they serve. Whether it is a different treatment provider, a housing organization, or a transportation company that will provide needed transportation to appointments and services, the organization will ideally foster a climate of respect and consideration for individuals impacted by trauma.

## Consideration of the Passage of Time

In thinking about the application of trauma research findings to service provision, it is meaningful to consider the impact of the passage of time on an individual’s recollection of the traumatic events in their life as well as the related emotional severity. There is a rich body of research studying how the passage of time affects memory related to traumatic events and their associated emotions (Brainerd et al., 2008; Bolton et al., 2006; Southwick et al., 1997; Strange et al., 2012). There is some evidence that there is memory amplification or enhancement of a traumatic event over time (Peace & Porter, 2004; Strange & Takarangi, 2015), yet there is also evidence supporting that memories of emotional events are relatively static (Conway et al., 1994) with the passage of time. These are important considerations in interpreting emotional severity findings related to experiences of trauma, such as reported by Greene and colleagues (2023) in relation to women with SUDs who are homeless or near homeless. Interms of translation to service provision, a clinician may be best situated to help a woman with an extensive trauma history by understanding how a patient currently feels and their current trauma, if applicable, as well as their recollections of past trauma and its’ related impact. Recall of past trauma severity is related to current symptom severity (Huh et al., 2017). Even if and when there is memory distortion, the current emotional recollection of the past trauma may be meaningful to the woman’s current treatment plan.

## Conclusion

Utilizing findings from descriptive studies that present rates of trauma experience particular to individuals served in mental health, SUD treatment and overall behavioral healthcare settings may improve service delivery and treatment experience and effectiveness. With committed leadership, behavioral healthcare organizations can bolster and nurture a trauma-informed workforce that is well-cared for to reduce the potential burden of secondary traumatic stress. Taking a research-informed approach, organizations can work to create a physically and emotionally safe organizational environment from the parking lot to the clinical session space to best engage and retain in healthcare treatment women with a history of trauma. At the clinical level, there are strategies to tailor treatment services in response to existing data related to trauma and other lived experiences of women with SUDs who are homeless or near homeless. Engaging these strategies could ultimately improve the effectiveness of behavioral health service provision.
